# Small-molecule targeting of translation initiation for cancer therapy

**DOI:** 10.18632/oncotarget.1186

**Published:** 2013-08-14

**Authors:** Bertal H. Aktas, Yuan Qiao, Esra Ozdelen, Roland Schubert, Sema Sevinc, Fred Harbinski, Luciano Grubissich, Samuel Singer, Jose A. Halperin

**Affiliations:** ^1^ Department of Medicine, Brigham and Women's Hospital, Boston; ^2^ Laboratory for Translational Research, Harvard Medical School, Boston; ^3^ Department of Surgery, Brigham and Women's Hospital, Boston

**Keywords:** CANCER, TERNARY COMPLEX, TRANSLATION, eIF2, UTR

## Abstract

Translation initiation plays a critical role in the regulation of cell growth and tumorigenesis. We report here that inhibiting translation initiation through induction of eIF2α phosphorylation by small-molecular-weight compounds restricts the availability of the eIF2·GTP·Met-tRNA_i_ ternary complex and abrogates the proliferation of cancer cells *in vitro* and tumor growth *in vivo*. Restricting the availability of the ternary complex preferentially down-regulates the expression of growth-promoting proteins and up-regulates the expression of ER stress response genes in cancer cells as well as in tumors excised from either animal models of human cancer or cancer patients. These findings provide the first direct evidence for translational control of gene-specific expression by small molecules *in vivo* and indicate that translation initiation factors are bona fide targets for development of mechanism-specific anti-cancer agents.

## INTRODUCTION

Translation initiation plays a critical role in both general and gene-specific regulation of gene expression and thereby in the control of cell proliferation, differentiation, and survival. Unrestricted translation initiation causes malignant transformation *in vitro* and likely plays a causative role in the genesis of some human cancers [1-3]. Consistently, translation initiation factors are over-expressed or otherwise activated in breast, lung, cervical, and hematologic cancers and predict poor prognosis [4-9]. In contrast, restricting translation initiation reverses transformed phenotypes *in vitro* and *in vivo* [10-13]. This experimental and clinical evidence indicates that translation initiation factors are potential targets for the development of mechanism-specific anti-cancer therapies. Indeed, an anti-sense oligonucleotide-based therapy targeting eukaryotic translation initiation factor (eIF) 4E inhibits tumor growth in animal models of cancer and has recently been evaluated in a phase-I-II human clinical trial[11]. However, whether small molecules can effectively target translation initiation *in vivo* has not been demonstrated. The studies reported here were designed to determine whether the eIF2·GTP·Met-tRNA_i_ translation initiation (ternary) complex is a bona fide target for the development of anti-cancer therapeutic agents.

Formation of the eIF2·GTP·Met-tRNA_i_ ternary complex is the first critical step in the translation initiation cascade. Upon assembly of the 80S ribosome at the initiation codon, GTP in the ternary complex is hydrolyzed, releasing the eIF2·GDP binary complex [14]. To initiate a new round of translation, eIF2·GDP must be converted to eIF2·GTP by eIF2B, the eIF2 guanine nucleotide exchange factor. Phosphorylation of the alpha subunit of eIF2 (eIF2α) on S51 increases the affinity of eIF2 for eIF2B and functionally converts eIF2 into a competitive inhibitor of eIF2B[15]. Because of the low eIF2B/eIF2 ratio, even partial phosphorylation of eIF2α limits the amount of the eIF2·GTP·Met-tRNA_i_ ternary complex and thereby inhibits translation initiation [16].

Restriction of the ternary complex by phosphorylation of eIF2α plays a critical role in the translational regulation of specific genes or gene clusters [17-22]. For example, the translation of mRNAs containing excessive and stable secondary structures, like those encoding for most oncogenic proteins, is preferentially down-regulated by phosphorylation of eIF2α at levels that would minimally affect translation of most housekeeping proteins encoded by mRNAs with simple 5'UTRs [23-29]. Paradoxically, the translation of a subset of mRNAs with particular features in their 5'UTRs, like the one coding for activating transcription factor-4 (ATF-4), is more efficient under conditions that limit the amount of the ternary complex [30, 31]. Increased translation of ATF-4 results in the transcriptional activation of its target genes including the chaperone BiP and the pro-apoptotic transcription factor CHOP [32].

We previously identified three chemically distinct small molecular weight compounds, Clotrimazole (CLT), eicosapentaenoic acid (EPA), and troglitazone (TRO), that cause phosphorylation of eIF2α in non-transformed cell lines [18, 20, 22, 33]. We used these compounds as molecular probes to determine whether restriction of the ternary complex via phosphorylation of eIF2α can be achieved pharmacologically for cancer treatment.

We report here that CLT, EPA, and TRO cause phosphorylation of eIF2α, restrict the amount of the ternary complex, and thereby inhibit translation initiation in a wide variety of cancer cell lines. In experimental cancer models, the compounds abrogate xenograft tumor growth and increase the mean survival time of p53^−/−^ mice. Abrogation of tumor growth is associated with phosphorylation of eIF2α, reduced availability of the ternary complex, down-regulation of oncogenes, and up-regulation of ATF-4-dependent genes. Importantly, we show that administration of TRO to liposarcoma patients caused phosphorylation of eIF2α and increased expression of the ATF-4-dependent protein BiP. These findings provide the first direct *in vivo* evidence that the ternary complex can be pharmacologically targeted for cancer therapy.

## RESULTS AND DISCUSSION

### CLT, EPA, and TRO inhibit cancer cell proliferation

We determined the effect of CLT, EPA, and TRO on the proliferation and/or survival of cancer cell lines by sulforhodamine B cell proliferation assay (SRB). Table 1 presents the IC50 of each compound for the cancer cell lines tested. The three molecular probes inhibited in a dose-dependent manner the proliferation of a wide variety of cancer cell lines, including several multiple drug-resistant ones.

**Table 1:** Effect of CLT, EPA, and TRO on Ca++ metabolism and proliferation in cancer cells

	CLT			TRO			EPA		
Cell line	IC50^1^	Ca^++^ release^2^	SOC close^2^	IC50*	Ca^++^ release^2^	SOC close^2^	IC50*	Ca^++^ release^2^	SOC close^2^
KLN HeLa Calu-6 MCF-7 ACHN DU-145 U118MG HCT 15 SK-OV3 SK-MEL 28 HTB 174 CRL 1933 SV480 HCT 116 HT 29 CRL 231 A549 MMRU HEP-G2	5 3 1 2 7 7 5 2 12 3 1.5 10 3.4 3 7 2 3 2 5	Y Y Y Y Y Y Y Y N Y Y Y ND ND Y ND Y Y Y	Y Y Y Y Y Y Y Y N Y Y N ND ND ND ND Y Y Y	8 12 15 10 18 17 34 12 17 30 20 15 14 8 20 26 8 ND 23	Y Y ND Y Y Y Y ND N ND Y Y ND ND Y ND Y ND Y	Y Y ND Y Y Y N ND N ND N ND ND ND N ND Y ND ND	20 10 40 43 62 36 36 47 96 46 79 ND ND ND 72 ND 54 36 ND	Y Y Y Y Y Y Y ND N ND Y ND ND ND Y ND Y Y ND	Y Y Y Y ND Y Y ND N ND N ND ND ND ND ND Y Y ND

1 Concentration of drug that inhibits cell growth by 50%.

2 Y = yes, N = no, ND = not done

### CLT, EPA, and TRO deplete internal Ca++ stores in cancer cells

We have previously reported that in NIH 3T3 cells, CLT, EPA, and TRO release Ca^++^ from internal stores and simultaneously inhibit their refilling through store-operated Ca^++^ channels (SOC) [18, 20, 22, 33]. This causes phosphorylation of eIF2α and inhibition of translation initiation [16, 20, 22]. To determine whether inhibition of cancer cell proliferation by CLT, EPA, and TRO is mediated by their effect on intracellular Ca^++^ homeostasis, we measured cytosolic Ca^++^ in Fura-2–loaded cancer cells. Table 1 shows that in most cancer cells, particularly those sensitive to their anti-proliferative effect, CLT, EPA, and TRO released Ca^++^ from internal stores – as indicated by an increased cytosolic Ca^++^ in Ca^++^-free media. To determine whether CLT, EPA, and TRO inhibit SOC in cancer cells, we first challenged Fura-2–loaded cancer cells with thapsigargin (TG), a specific inhibitor of SERCA-ATPase [34, 35]. Treatment with TG in media containing Ca^++^ results in sustained elevation of cytosolic Ca^++^, a functional expression of capacitative Ca^++^ influx through SOC. Addition of CLT, EPA, or TRO after TG closes SOC in most cancer cell lines, as indicated by a sharp reduction of cytosolic Ca^++^ towards the baseline levels (Table 1). To document directly whether the compounds release Ca^++^ from the ER stores, as suggested by the FURA-2 experiments, we generated stable cell lines that express calcium-indicator cameleon proteins targeted to the ER[36]. These experiments formally demonstrate that CLT, EPA, and TRO partially deplete ER Ca^++^ stores (see Supplemental Results and in Supplemental Figure 1A for details).

**Figure 1:**
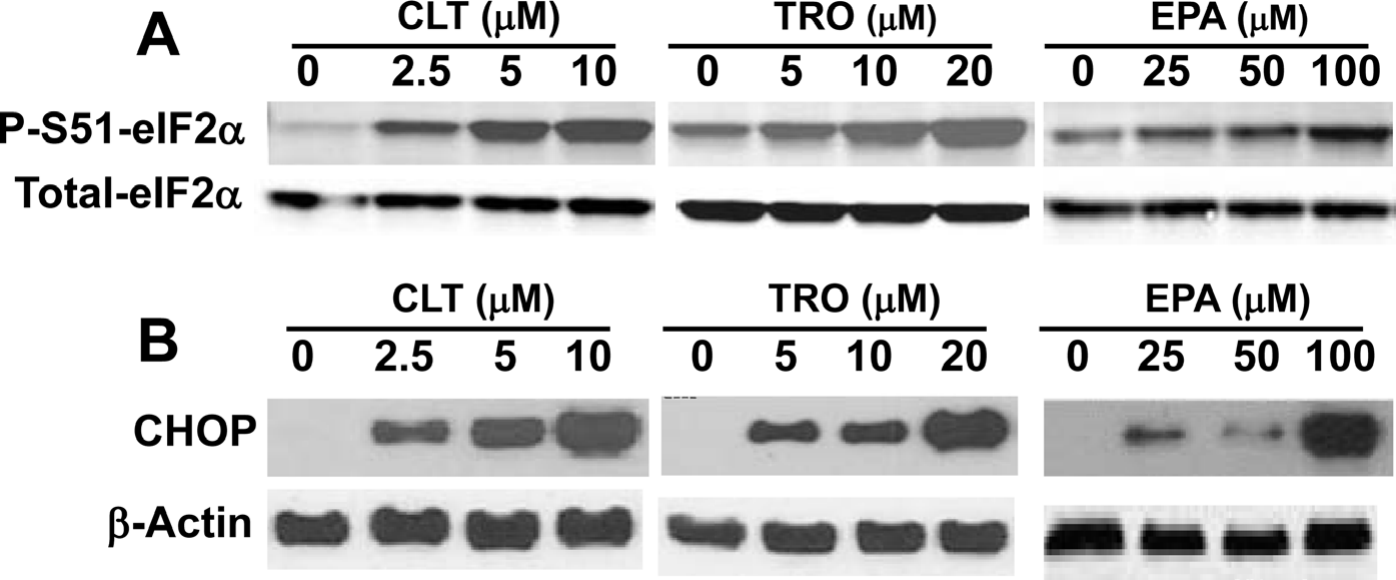
CLT, EPA, and TRO cause eIF2α phosphorylation and induce CHOP expression in KLN cells

KLN cells were incubated with indicated concentrations of CLT, EPA, and TRO for 2 (A) or 8 (B) hours, the cells were lysed, and equal amounts of protein were separated by SDS-PAGE and blotted with anti-PS51-eIF2α, anti-total eIF2α (2 hours treated samples, A) or anti-CHOP and anti-β-actin (8 hours treated samples, B) specific antibodies.

Taken together, the data presented in Table 1 and Supplemental Figure 1A demonstrate that CLT, EPA, and TRO partially deplete ER- Ca^++^ stores in a variety of human cancer cell lines. Because the effect on intracellular Ca^++^ was similar in all cancer cell lines sensitive to the anti-proliferative effect of the compounds, further mechanistic and in vivo studies were conducted in selected cancer cell lines.

### CLT, EPA, and TRO induce phosphorylation of eIF2α and inhibit translation initiation in cancer cells

Extensive experimental evidence shows that partial depletion of ER Ca^++^ stores induces phosphorylation of eIF2α [18, 20]. As expected from their effect on ER Ca^++^, CLT, EPA, and TRO caused strong phosphorylation of eIF2α in KLN squamous cell carcinoma cells (Figure 1A), as well as in MCF-7 breast cells and DU145 prostate cancer cells (data not shown).

To determine the functional consequences of eIF2α phosphorylation, we took advantage of the well-documented translational up-regulation of ATF-4 and consequent induction of ATF-4-responsive genes that result from eIF2α phosphorylation, which reduces the amount of eIF2·GTP·Met-tRNAi ternary complex [32]. As shown in Figure 1B, CLT, EPA, and TRO strongly induce the expression of CHOP, a transcriptional target of ATF-4. These data suggest that CLT, EPA, and TRO reduce the amount of the ternary complex and thereby inhibit translation initiation in cancer cells.

### CLT, EPA, and TRO restrict formation of the ternary complex *in vitro* and *in vivo*

To confirm that CLT, EPA, and TRO restrict the abundance of the eIF2·GTP·Met-tRNAi ternary complex, we developed a quantitative cell-based assay whose read-out depends on the availability of this complex. The assay takes advantage of the striking differential effect that restriction of the ternary complex exerts on the translation of mRNAs. In particular, translation of a subset of mRNAs characterized by the presence of multiple upstream open reading frames (uORF) in their 5'UTR increases when the ternary complex is restricted, as characteristically represented by the mRNA encoding for ATF-4 and GCN2 [32, 37, 38]. Fusion of the 5' UTR of either mRNA to a reporter gene confers enhanced translation when the ternary complex is scarce [32, 38].

**Figure 2:**
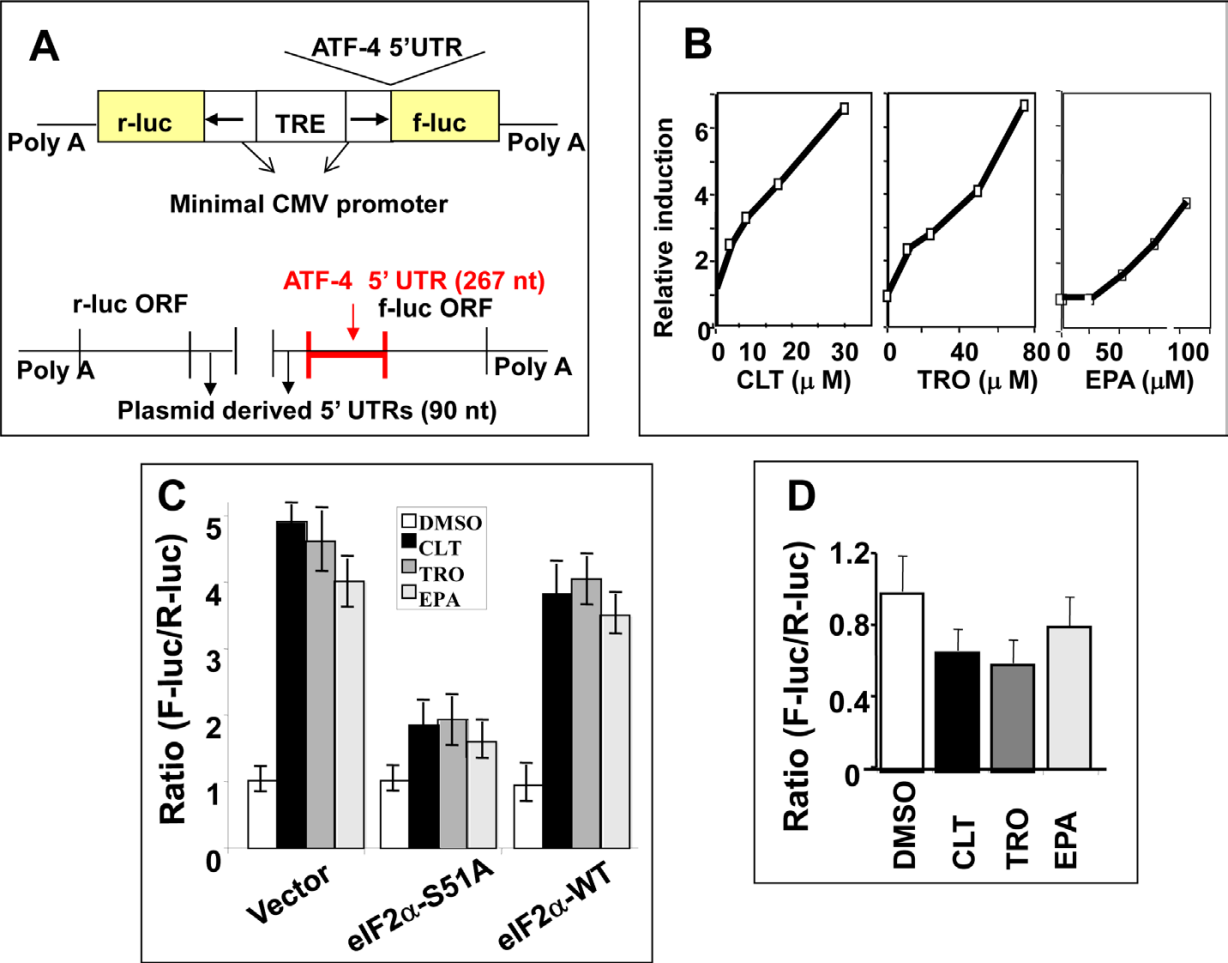
CLT, EPA, and TRO limit formation of the ternary complex

Assay development has been described elsewhere [39, 40]. Briefly, we constructed a bi-directional plasmid in which a common promoter/enhancer complex drives the transcription of firefly luciferase (F-luc) ORF fused to the 5'UTR of ATF-4, and of the renilla luciferase (R-luc) ORF fused to a simple 90-nucleotide 5'UTR derived from the plasmid (Figure 2A). The relative expression of each luciferase was established by the F-luc to R-luc ratio determined with a dual luciferase assay. In stably transfected KLN cells, CLT, EPA, or TRO increased the F-luc to R-luc ratio in a dose-dependent manner without affecting the ratio of the respective mRNAs (Figures 2B and 2C). These data indicate that CLT, EPA, and TRO increase the translation of the reporter F-luc mRNA fused to the 5'UTR of ATF-4. To establish the cause-effect relationship between phosphorylation of eIF2α and the increased translation of F-luc mRNA shown in Figure 1, we transiently co-transfected KLN cells with the bi-directional construct described in Figure 2A and an expression vector coding for either wild-type eIF2α (eIF2α-WT) or a constitutively active non-phosphorylatable eIF2α mutant (eIF2α-S51A). Figure 2D shows that co-transfection with the eIF2α-S51A mutant abrogated the increase in the F-luc to R-luc ratio observed in response to CLT, EPA, or TRO, while co-transfection with eIF2α-WT had no effect. Furthermore, a single nucleotide insertion that removes the second uORF in the reporter construct abrogated the translational up-regulation of F-luc induced by these agents (Figure 2E). Abrogation of F-Luc translational up-regulation by either non-phosphorylatable eIF2α or by deletion of one uORF demonstrates conclusively that CLT, EPA, or TRO reduces the availability of the ternary complex by inducing phosphorylation of eIF2α. Consistently, anti-cancer agents that do not cause eIF2α phosphorylation failed to up-regulate F-luc (see supplementary Table 1).

A) Schematic depiction of expression vector and its mRNA products utilized for establishment of a ternary complex availability assay. B) KLN cells stably transfected with this vector were incubated with CLT, EPA, or TRO for 24 hours, and reporter activities were determined by DLR (Promega, Inc.). Changes in the F-luc/R-luc ratio were expressed as multiples of the same ratio in vehicle-treated cells. C) Poly A plus mRNA was isolated from KLN cells in B and hybridized with probes specific to F-luc or R-luc mRNA. D) KLN cells were co-transfected with the bi-directional vector shown in Figure 2A and the eIF2α-WT or eIF2α-51A expression vectors. The F-luc/R-luc ratio in response to CLT, EPA, or TRO was measured by DLR assay as in B. E) A single nucleotide was inserted into the second uORF of the ATF-4 5'UTR, which eliminated the ORF2. Cells transfected with this mutant construct were treated with CLT, EPA, or TRO, as in 2B.

### CLT, EPA, and TRO inhibit translation initiation

**Figure 3:**
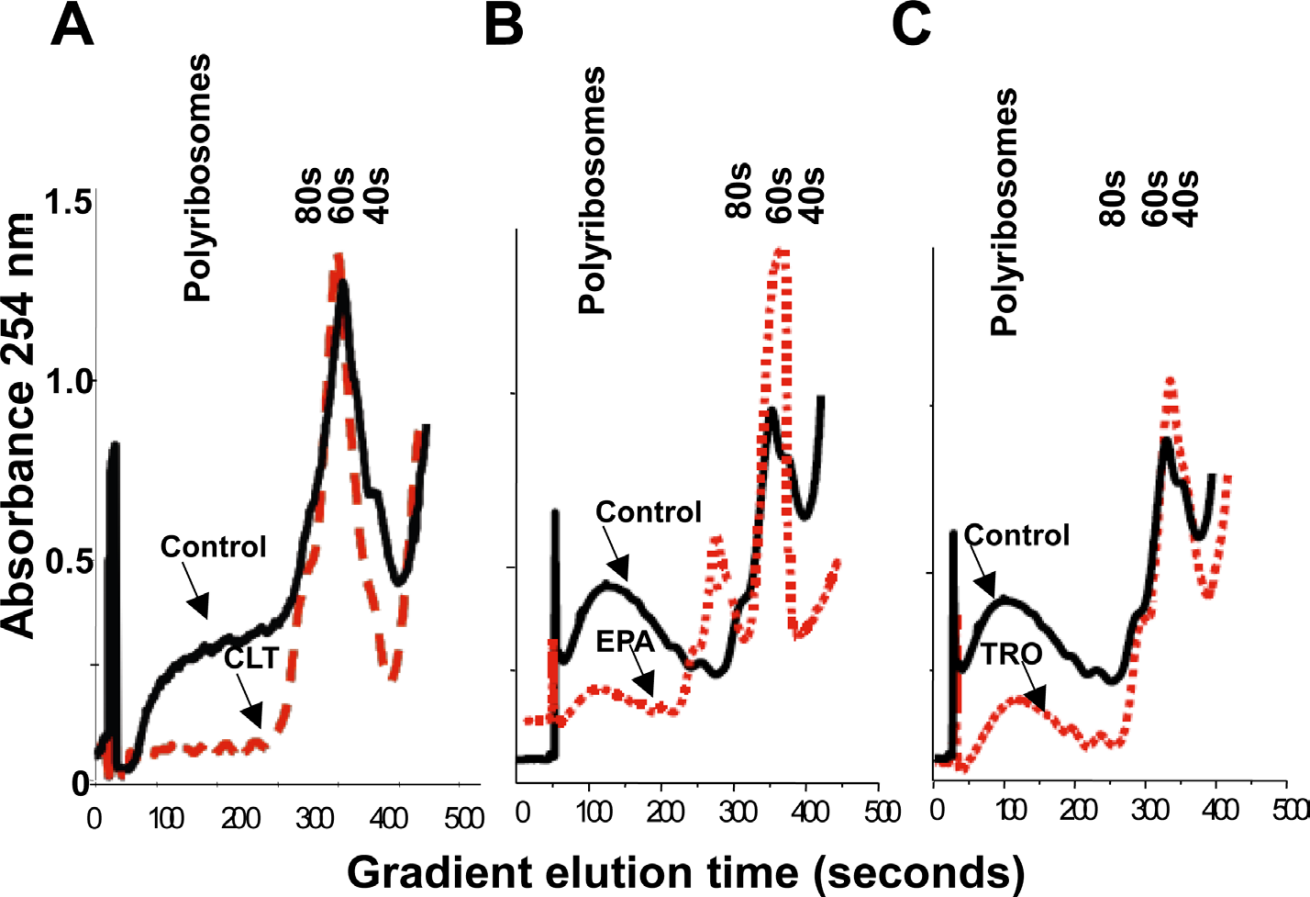
CLT, EPA, and TRO inhibit translation initiation in cancer cells

The results depicted in Figures 1 and 2 strongly indicate that CLT, EPA, and TRO inhibit translation initiation in cancer cells, a conclusion that can be tested experimentally by analyzing polysome profiles of cell lysates. When translation initiation is inhibited, polysome profiles shift from heavy toward lighter polysomes. As shown in Figure 3A-C, the polysome profiles of CLT, EPA, and TRO-treated KLN cells shifted from heavy to light polysomes and free ribosomal subunits, demonstrating that the three agents inhibit translation initiation in cancer cells. This conclusion was confirmed in pulse-labeling experiments demonstrating that CLT, EPA, and TRO inhibit protein synthesis in different cancer cell lines (Supplemental Figure 1B).

KLN cells were incubated with CLT (A, 15 μM), EPA (B, 50 μM), or TRO (C, 30 μM) for 2 hours, lysed, and an equal amount of OD 260 material was separated by sucrose density gradient centrifugation. The gradient was eluted from the bottom with constant monitoring at 254 nm.

### CLT-, EPA-, and TRO-induced cell cycle arrest in cancer cells is mediated by phosphorylation of eIF2α

To determine the effect of CLT, EPA, and TRO on cell cycle progression in cancer cells, we treated exponentially growing KLN cancer cells with these three agents, stained them with propidium iodide, and analyzed them by FACS for cell cycle distribution and apoptosis. CLT, EPA, and TRO blocked the KLN cancer cell cycle in G1 with no apparent effect on the proportion of apoptotic cells (Supplemental Figure 2). This cell cycle arrest is mediated by phosphorylation of eIF2α on S51, because it was abrogated in cells transiently transfected with the non-phosphorylatable eIF2α-S51A mutant (Table 2).

**Table 2:** Effect of CLT, EPA, and TRO on cell cycle progression of eIFα-51A/GFP or eIF2α-WT/GFP transfected KLN-mouse carcinoma cells* (the percentage of cells in each cell cycle stage)

Transfected Vector	Treatment			
	DMSO	CLT	EPA	TRO
eIF2α-WT/GFP	G1: 47 S: 25 G2: 28	G1: 65 S: 19 G2: 16	G1: 61 S: 22 G2: 17	G1: 67 S: 21 G2: 12
eIF2α-51A/GFP	G1: 45 S: 26 G2: 29	G1: 52 S: 27 G2: 21	G1: 49 S: 29 G2: 22	G1: 53 S: 24 G2: 23

* KLN cells transiently transfected with either GFP-eIF2α-51A (A, B) or GFP-eIF2α-WT (C, D) were treated with CLT, EPA, or TRO, sequentially fixed with 3% paraformaldhyde and 70% ethanol, stained with propidium iodide, and cell cycle distribution of GFP-expressing cells was determined by FACS analysis.

### CLT, EPA, and TRO cause phosphorylation of eIF2α, induce expression of BiP, and down-regulate expression of cyclin D1 in tumors

**Figure 4:**
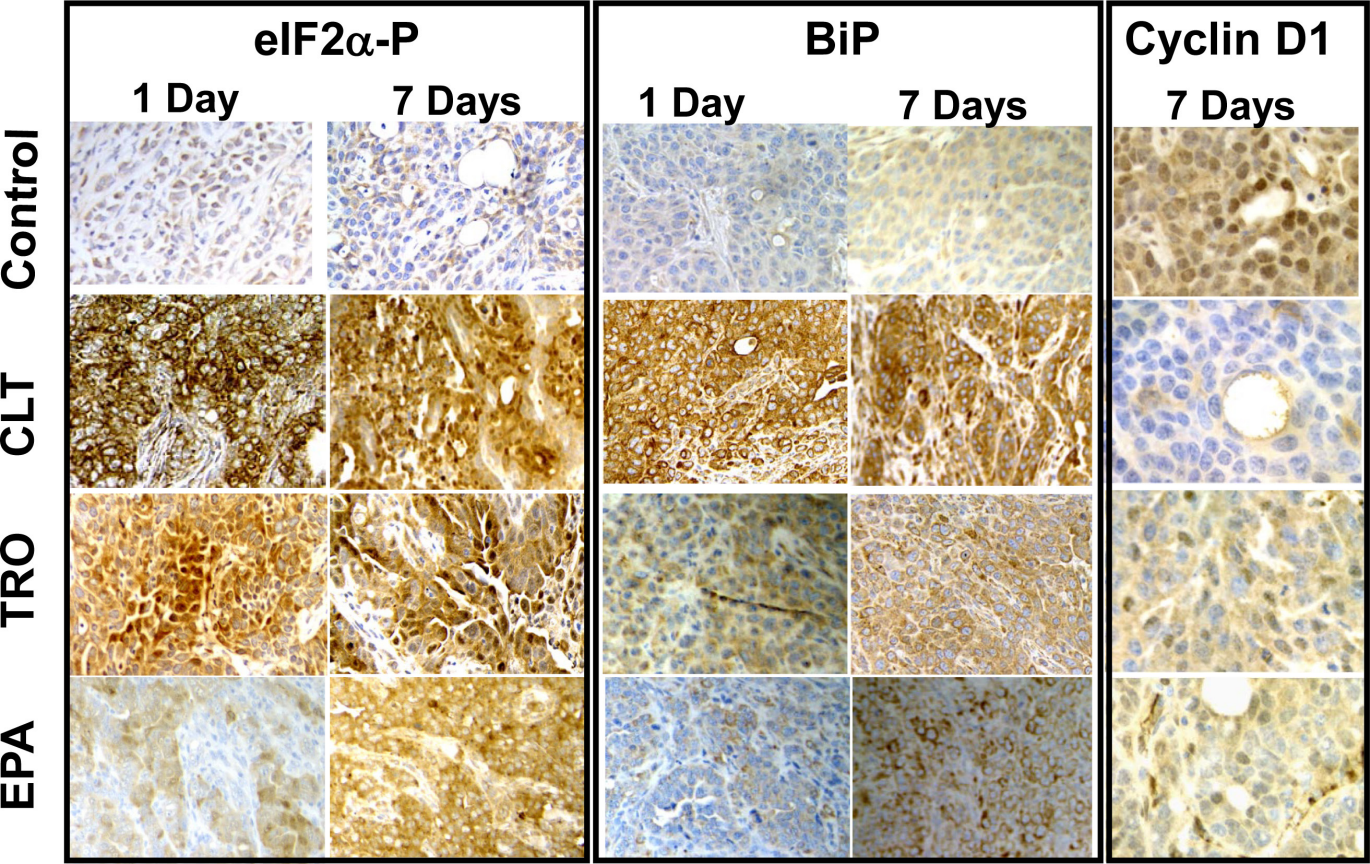
CLT, EPA, and TRO cause phosphorylation of eIF2α and induction of BiP and suppress expression of cyclin D1 in KLN tumors

To determine directly whether phosphorylation of eIF2α in tumors can be achieved pharmacologically, we implanted KLN cells intradermally in mice to form orthotopic-syngenic tumors. Mice carrying small (2-4 mm in diameter) KLN tumors were treated for either one or seven days orally with CLT (120 mg/kg), EPA (2.5 g/kg), TRO (350 mg/kg), or the respective vehicles. Staining of excised tumors with anti-PS51-eIF2α−specific antibodies showed that all three agents induced significant and sustained phosphorylation of eIF2α, providing the first experimental evidence that phosphorylation of eIF2α in tumors can be achieved pharmacologically (Figure 4). Consistently, in tumors from treated animals, all three agents increased the expression of BiP, another ATF-4 dependent surrogate marker for depletion of the ternary complex that is induced by phosphorylation of eIF2α[30-32, 41]. CLT, EPA, and TRO also down-regulated the expression of cyclin D1 in the tumors excised from treated mice (Figure 4). This finding is consistent with depletion of the ternary complex, which preferentially affects translation of mRNAs containing highly structured 5'UTRs, including those encoding for most oncogenic proteins such as cyclin D1[18, 20, 22, 42]. To confirm restriction of the ternary complex in the tumors, we injected mice intradermically with the engineered KLN cells shown in Figure 2B and treated tumor-bearing mice with CLT (120 mg/kg/day) or vehicle for two days. Supplemental Figure 3 shows that CLT significantly increased the F-luc to R-luc ratio in the tumors from CLT-treated mice. Taken together, the experiments described above demonstrate that restriction of the ternary complex and inhibition of translation initiation in tumors can be pharmacologically achieved.

Mice with small KLN tumors were treated with CLT (120 mg/kg/day), EPA (2.5.g/kg/day), TRO (350 mg/kg/day), or vehicle for one day (left panels) or seven days (right panels). The tumors were excised and stained with antibodies specific to PS51-eIF2α, BiP, or cyclin D1 and counter-stained with hematoxyline.

### CLT, EPA, and TRO inhibit tumor growth in animal models

**Figure 5:**
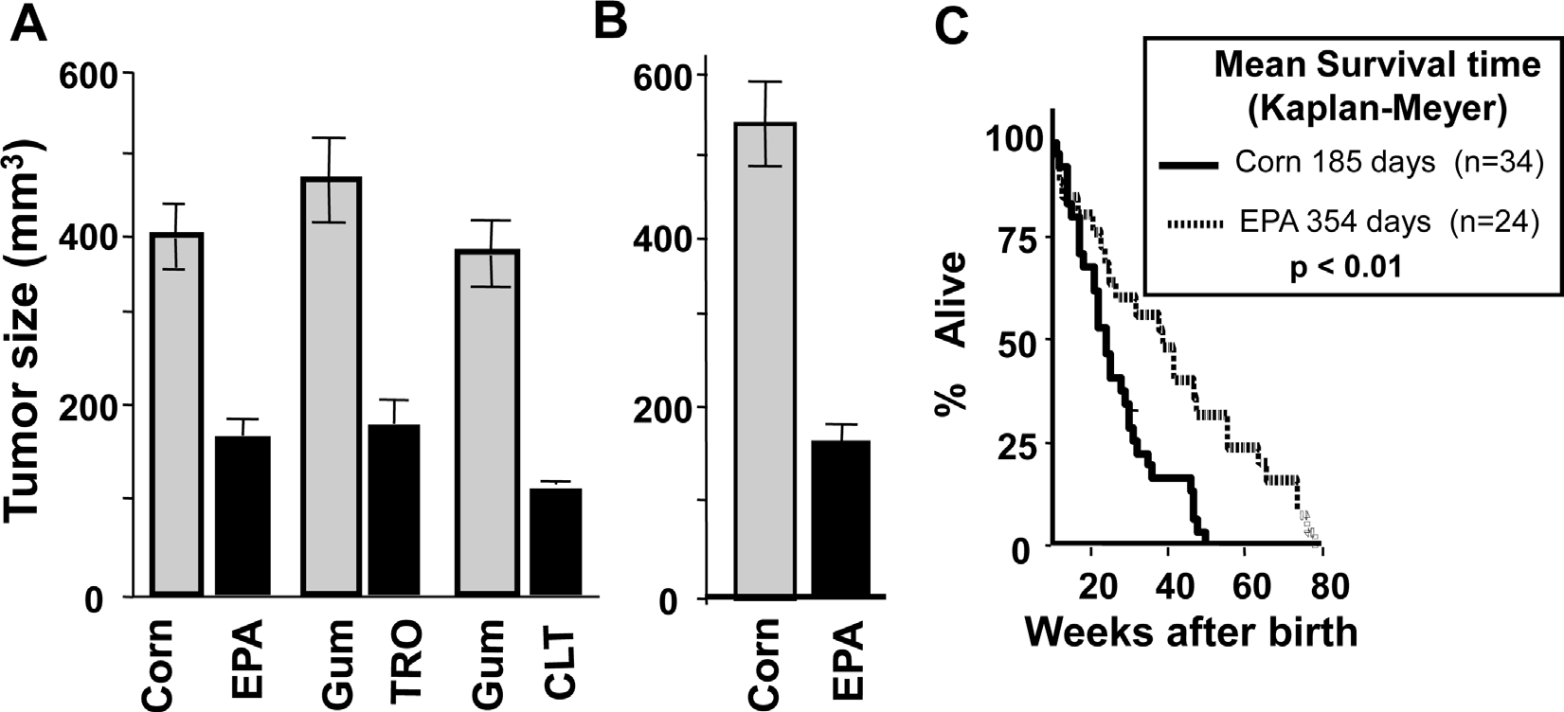
CLT, EPA, and TRO inhibit the growth of tumors and EPA extends the life expectancy of p53−/− mice

To determine the functional consequence of reducing the amount of the ternary complex in tumors, we treated KLN-cell tumor-bearing mice orally with CLT (120 mg/kg), EPA (2.5 g/kg), or TRO (350 mg/kg) daily for 5 weeks. Control mice for CLT or TRO received gum arabica (the vehicle used to dissolve the drugs), while control mice for EPA received equicaloric amounts of corn oil. As shown in Figure 5A, CLT, EPA, and TRO significantly reduced the growth of KLN cell tumors, demonstrating that phosphorylation of eIF2α and restriction of the ternary complex is associated with inhibition of tumor growth.

The observation that EPA induces phosphorylation of eIF2α and inhibition of KLN-tumor growth *in vivo* is remarkable and has major public health implications, because EPA, the main n-3 fatty acid in marine fish oil, is a component of the human diet. Indeed, some human population studies suggest that consumption of diets rich in EPA may reduce cancer risk [43-45]. For this reason, we probed further the effect of EPA on tumor growth and progression in a DU-145 human prostate cancer xenograft and in a genetically engineered p53^−/−^ mouse cancer model. Treatment with EPA significantly reduced the growth of DU-145 human prostate cancer xenograft tumors (Figure 5B). In p53^−/−^ mice, oral administration of EPA significantly extended the mean survival time from 185 to 360 days (p<0.005, Figure 5C). This experiment is remarkable, because p53 is the most commonly mutated or deleted gene in human cancers, and p53^−/−^ mice develop various cancers with 100% penetrance and all die before 40 weeks of age.

A) DBA/2J mice were injected with 2.5 × 10^6^ KLN cells, the animals were randomly assigned to the treatment and control groups after establishment of tumors and treated with 120 mg/kg/day CLT, or 350 mg/kg/day TRO, both in gum arabica, or EPA (2.5 g/kg, administered as fish oil concentrate). Gum arabica-treated mice were used as a control for CLT and TRO, and corn oil-treated mice were used as a control for EPA treatment. The experiments were carried out in pairs (CLT vs. gum arabica, TRO vs. gum arabica, EPA vs. corn oil) with 15 mice per group. Tumor dimensions were determined with electronic calipers, tumor volume was calculated, and the results were analyzed by Student's t-test. B). Male nude mice were injected with 1×10^6^ human DU145 prostate cancer cells, and mice with visible tumors were fed fish oil- or corn oil-enriched diets for five weeks. C) p53^−/−^ mice were treated with either EPA (2.5 g/kg administered as fish oil concentrate) or an equicaloric amount of corn oil starting at four weeks of age; the time of death was recorded. The data were analyzed by Kaplan-Mayer test (p<0.01).

### Troglitazone causes phosphorylation of eIF2α and induction of BiP expression in human cancers

**Figure 6:**
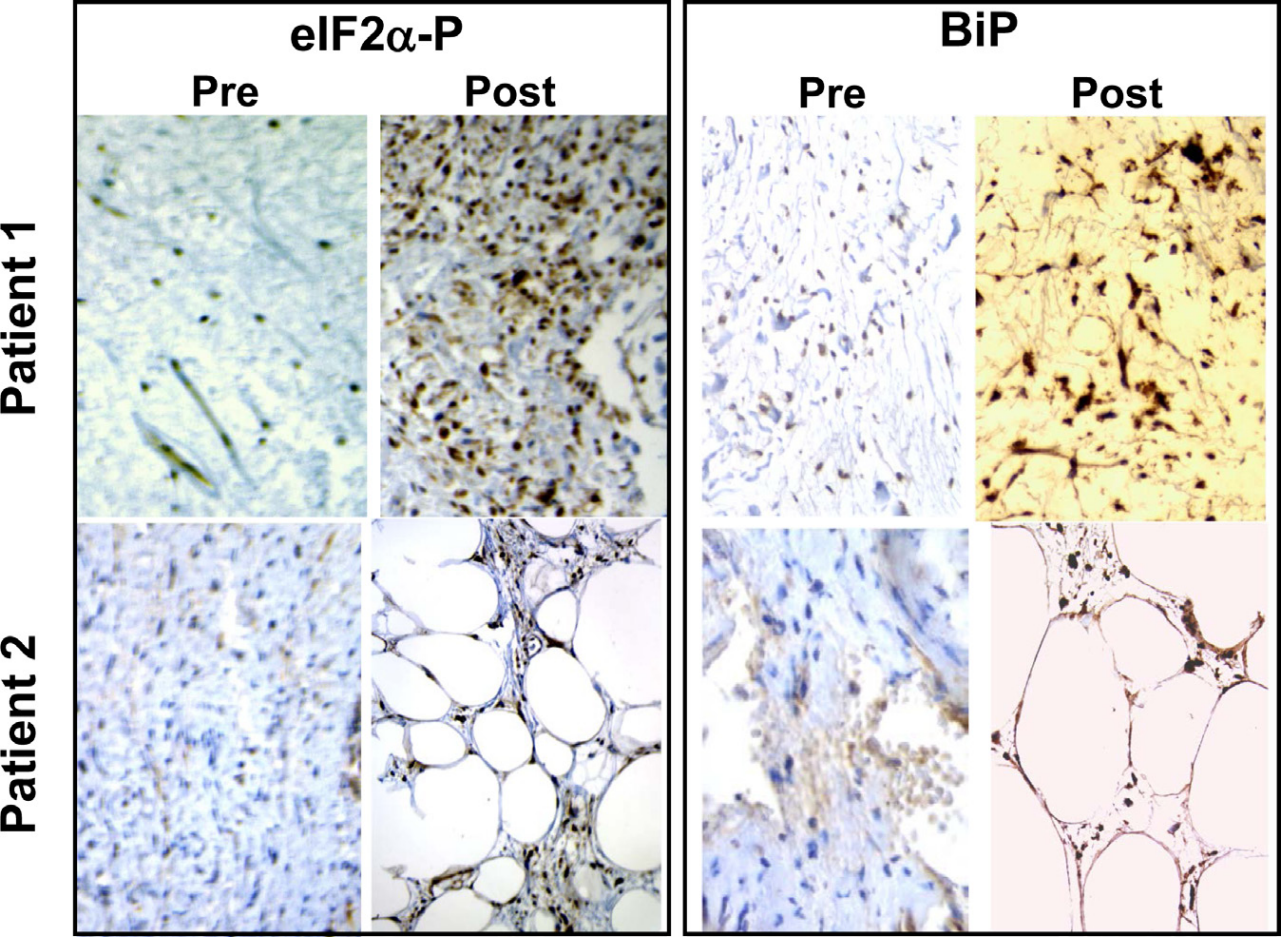
TRO induces phosphorylation of eIF2α in human liposarcoma tumors

While this work was in progress, we became aware of a clinical trial in which TRO was administered to liposarcoma patients between a diagnostic biopsy and the surgical excision of the tumor [46]. We obtained paraffin-embedded pathology samples from these patients and stained them with anti-PS51-eIF2α- and BiP-specific antibodies. Figure 6 shows that treatment with TRO caused a remarkable increase in eIF2α phosphorylation and BiP expression in the excised human liposarcoma tumors compared with the pre-treatment biopsy.

Clinical and experimental studies indicate that dysregulation of translation initiation plays a role in malignant transformation and tumor progression, highlighting the potential of therapeutics that exert antitumor and/or chemopreventive effects by selectively targeting the translation initiation cascade[1, 47-49]. The findings reported in this manuscript provide conclusive evidence that translation initiation can be pharmacologically targeted *in vivo* with small molecules, a critical prerequisite for the development of translation initiation-specific anti-cancer agents. Specifically, we demonstrate that 1) pharmacological agents that induce phosphorylation of eIF2α and reduce the ternary complex in vitro similarly phosphorylate eIF2α and reduce the ternary complex in animal and human tumors, and 2) phosphorylation of eIF2α in vivo is associated with suppression of tumor growth and extension of life expectancy in various animal cancer models. Direct *in vivo* demonstration of the cause-effect relationship studies between induction of eIF2α phosphorylation and tumor by replacing endogenous eIF2α with recombinant eIF2α-S51A mutant could not be carried out despite our attempts because tumors formed by these cells failed to grow well after an initial phase. Similar results have been reported by others [50].

Together, the *in vivo* experiments depicted in Figure 5 and 6 indicate that pharmacological inhibition of translation initiation can be used for cancer treatment and perhaps prevention in high-risk populations. Second generation of translation initiation inhibitors being developed as well as progress in resolving the structure of translation initiation factors and their regulators should accelerate development of translation initiation inhibitors for cancer therapy [39, 51, 52]. Furthermore, since EPA is the main component of marine fish oils and can be readily administered to humans with minimal or no side effects, our studies provide a strong favorable rationale and the analytical tools for human clinical trials aimed at evaluating whether translation initiation inhibitors that induce phosphorylation of eIF2α would reduce either recurrence in cancer patients who are in remission or the incidence of cancer in high-risk populations.

Biopsy samples of liposarcomas obtained before treatment (left panels) and tumor samples of the same patients after treatment with TRO (right panels) in a clinical trial conducted independently of our work[46] were stained with antibodies specific to PS51-eIF2α or to BiP.

## METHODS

### Ethics Statement

All animal studies in this report are carried out per Harvard Medical School Standing Committee on Animals, IACCUC, that oversees protocols involving animals per approved protocol #03151. The end point in studies with p53−/− and p53+/+ was lifespan. Animals were observed daily and weighed twice weekly. They were sacrificed in they had locomotion or breathing difficulties or lost more than 15% of body weight or appeared cachectic. In all other studies, animals were maintained for total of four weeks after the start of treatment. They were observed at least three times a week, any animal that had tumor volume greater than 15% of body weight or lost 15% body weight or had breathing or locomotion difficulties or other signs of pain or suffering per approved protocol were sacrificed. All animals were given analgesic (bprunex) as needed.

Human samples are retrospective discarded pathological materials with no identifiers. Partners HealthCare Human Research Committee, the Partners Internal Review Board for Human Studies approved the collection of samples as de-identified discarded pathological materials not requiring consent.

### Cell growth assay

Cell growth was measured by the SRB assay as described in [22].

### Measurements of free cytosolic and ERCa++

Free intracellular Ca^++^ was measured using Fura-2, AM (Molecular Probes, Eugene, OR) as described by Clementi et al. [53]. Fluorescence was monitored with a Photon Technology International dual-wavelength spectrofluorometer (PTI, Monmouth Junction, NJ). Excitation was at 340/380 nm, and emission at 510 nm. For measurement of ER Ca^++^, we established stable cell lines expressing ER-targeted cameleon proteins [36, 54] and monitored ER Ca^++^ using the PTI dual-wavelength spectrofluorometer.

### Polysome profiles

Polysome profiles were obtained by sucrose density gradient centrifugation as described in[55].

### Stable and transient transfection

Cells were seeded at the density of 10^5^ in 60-mm (stable transfection) or 10^6^ cells per 150-mm (transient transfection) plates and transfected one day later using the Qiagen transfectamine transfection kit. For selection of stable cell lines, transfected cells were transferred to 100-mm plates and selected with appropriate antibiotics.

### Cell cycle analysis

Untransfected KLN cells were fixed with ethanol (70%), stained with propidium iodide (50 μg/ml PI with 20 μg/ml RNAse A) for 30 minutes, and analyzed by FACS[56]. Cells transiently transfected with GFP-eIF2α-51A or eIF2α-WT were fixed with 3% paraformaldhyde, washed twice with PBS, fixed again with 70% methanol, and stained with PI as described above. Cell cycle distribution of GFP-expressing cells was analyzed by FACS.

### Western blotting

Cell extracts were separated by SDS-PAGE and probed with anti-phosphoserine-51-eIF2α (PS51-eIF2α), anti-total eIF2α-specific antibodies (PS51-eIF2α) (Biosource International, Hopkinton, MA), anti-CHOP, or anti β-actin as described [56].

### Animal studies

KLN squamous cell carcinoma cells were injected intradermically (2.5 × 10^5^ cells in 0.1 ml of PBS) into 6-week-old female DBA/2J mice (Jackson Laboratories, Bar Harbor, ME). Four days after implantation, tumor-bearing mice were randomized into control and treatment groups and then treated with the vehicle or CLT (120 mg/kg in gum arabica), TRO (400 mg/kg in gum arabica), or EPA (2.5 g/kg, lipid concentrate) daily by gavage. Control mice were treated with the respective vehicle alone. Tumor volumes were calculated as in [20] and results analyzed by Student's t-test. DU145 human prostate cancer cells were subcutaneously injected to nude mice that were fed a diet containing either 20% corn oil or 1% corn oil and 19% menhaden oil, starting five days after inoculation of tumors. Tumor volumes were calculated[20] and results analyzed by Student's t-test. p53^−/−^ mice were administered either EPA (2.5 g/kg in fish oil refined for high content of EPA) or equicaloric corn oil daily by gavage until the death of the last animal. Life expectancies of control and EPA-treated mice were compared by Kaplan-Meyer test.

The end point in studies with p53−/− and p53+/+ was lifespan. Animals were observed daily and weighed twice weekly. They were sacrificed in they had locomotion or breathing difficulties or lost more than 15% of body weight or appeared cachectic. In all other studies, animals were maintained for total of four weeks after the start of treatment. They were observed at least three times a week, any animal that had tumor volume greater than 15% of body weight or lost 15% body weight or had breathing or locomotion difficulties or other signs of pain or suffering per approved protocol were sacrificed. All animals were given analgesic (bprunex) as needed.

### Immunocytochemistry

Formalin-fixed, paraffin-embedded tumor sections were immunostained with anti-PS51-eIF2α, anti-BiP, and anti-cyclin D1 antibodies (Santa Cruz Biotechnology, Santa Cruz, CA), and counter-stained with hematoxylin.

### Dual luciferase assay

Cells or minced tumors expressing F-luc and R-luc were lysed and the extracts assayed with a glow-type dual luciferase assay kit, (Promega, Inc., Madison, WI).

## Supplementary Figure and Tables




