# Supplementary material for: Small-molecule targeting of translation initiation for cancer therapy
Source: Oncotarget. 2013 Aug 14;4(10):1606–17. doi: 10.18632/oncotarget.1186 (PMC3858549; doi:10.18632/oncotarget.1186)
Supplement: Supplementary file 1 [file oncotarget-04-1606-s001.pdf]

Small-Molecule Targeting of Translation Initiation for Cancer Therapy - Aktas et al

Supplemental Results.

Release of calcium from internal stores and concomitant closing of SOC suggest that these compounds inhibit translation initiation by partial depletion of endoplasmic reticulum (ER)-Ca++ stores. To determine directly whether CLT, EPA, and TRO partially deplete ER-Ca++ stores, we constructed stable cell lines that express ER-targeted cameleon proteins (1, 2). Cameleons are the fusion of calmodulin and calmodulinbinding peptide (CBP), flanked on each side by cyan fluorescent protein (CFP) and yellow fluorescent protein (YFP). In the absence of Ca++, fusion protein is in a relaxed configuration; therefore CFP and YFP are distant from each other. Addition of Ca++ increases the affinity of calmodulin for CBP, brings YFP and CFP into close proximity, and allows for fluorescence resonance energy transfer (FRET) (1, 2). Because the Ca++ concentration in the ER is high, we expected to see a large FRET signal in untreated cells. In contrast, decreasing the ER Ca++ concentration would attenuate the FRET signal, as documented with TG (Supplemental Figure 1A). Supplemental Figure 1A shows that addition of CLT, EPA, or TRO to stable cell lines expressing ER-targeted cameleon proteins decreased FRET with a time course comparable to the effect of these probes on cytosolic calcium in Fura-2 loaded cells. Comparison with the effect of TG shows that CLT, EPA, and TRO induce a substantial depletion of calcium stored in the ERcompartment.

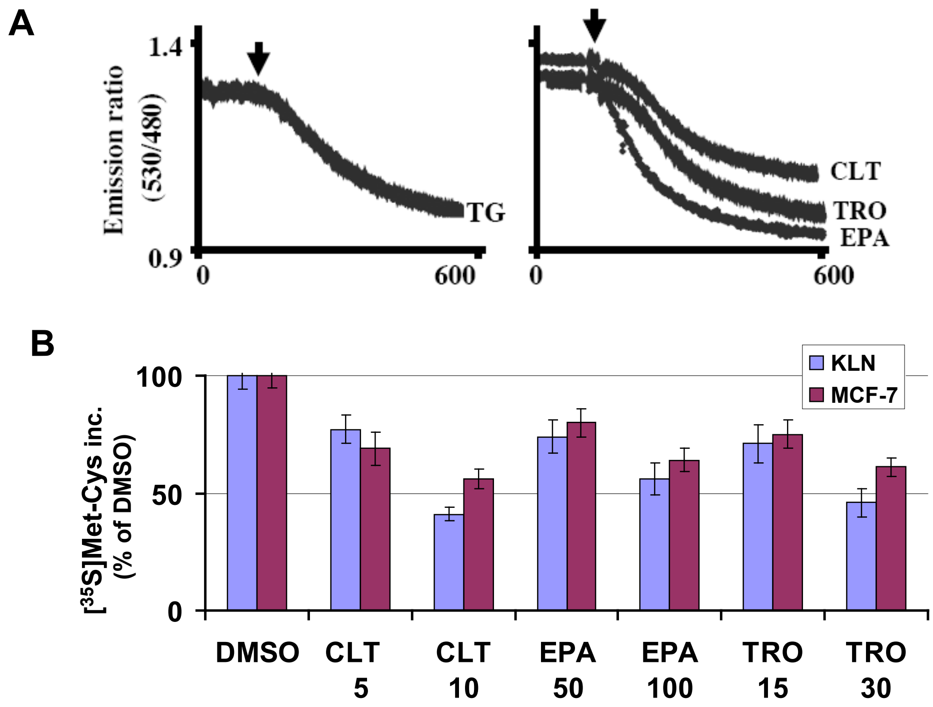

Supplemental Figure 1: CLT, EPA, and TRO partially empty ER Ca++ stores and inhibit protein synthesis A.) Cells were transfected with ER-targeted cameleon proteins and stable cell lines were obtained. These cells were treated with TG, CLT, EPA, or TRO. ER Ca++ and ER Ca++ was monitored by exciting cells at 445 nM and measuring ratio of emission at 480 and 530 nM (FRET) using the PTI dual-wavelength spectrofluorometer. B). CLT, EPA, and TRO inhibit protein synthesis in cancer cells. MCF-7 human breast cancer and KLN mouse squamous carcinoma cells were pulse labeled with [35S]methionine-cysteine (100μCi/ml) in the presence of the indicated concentrations of CLT, EPA, or TRO for 30 minutes. Cell lysates were blotted on nitrocellulose filter and washed 3x with 5% TCA before being counted in a scintillation counter. Incorporation of radiolabel per μg of proteins was quantified and normalized for vehicle-treated cells.

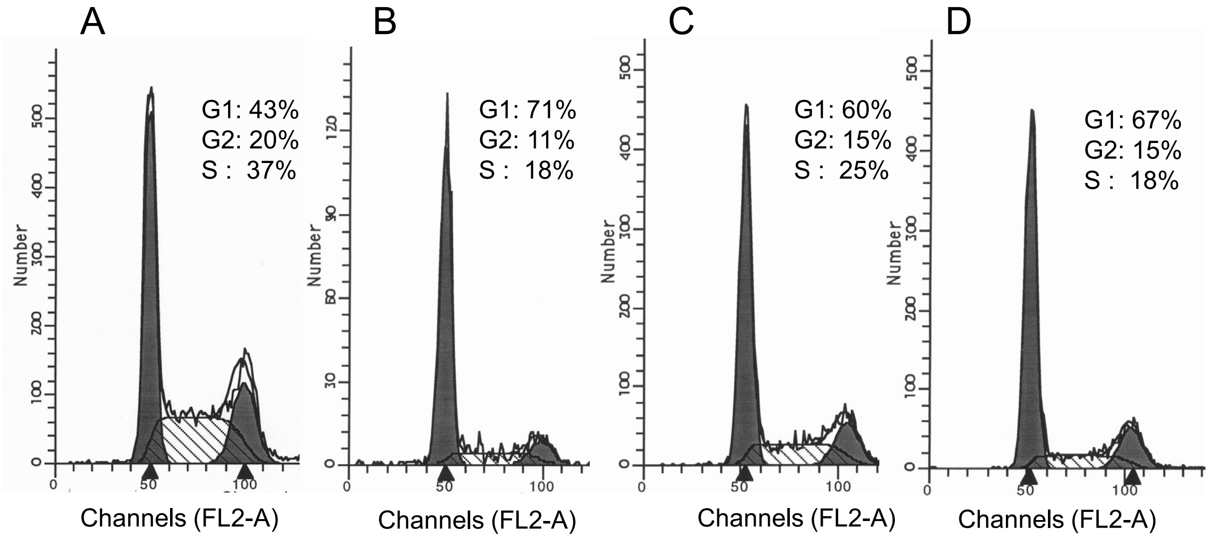

Supplemental Figure 2: CLT, EPA, and TRO arrest KLN cancer cells in G1. KLN cells were incubated with vehicle (A), CLT (15 µM, B), EPA (100 µM, C), or TRO (30 µM, D) for 48 hours, fixed, and stained with propidium iodide. Cells were analyzed for apoptosis and cell cycle distribution by FACS analysis.

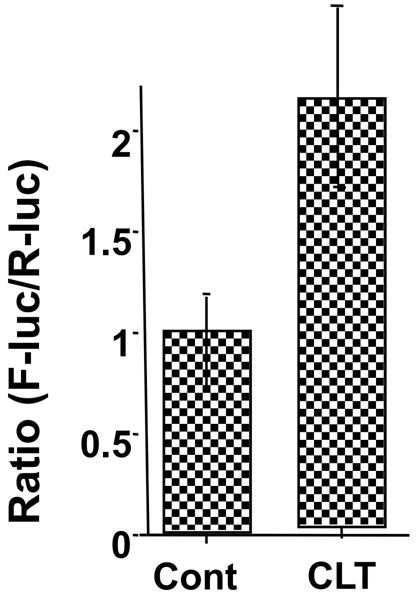

Supplemental Figure 3: CLT depletes eIF2.GTP.met-tRNAi ternary complex in the tumors. 2x106 cells described in 2B were subcutaneously injected into nude mice to form tumors; mice with small (2-5-mm diameter) tumors were treated for two days by gavage with either CLT (120 mg/kg/day) or vehicle, tumors were excised, and the activity of F-luc and R-luc was determined by DLR.

SUPPLEMENTAL REFERENCES

1. Miyawaki, A., Llopis, J., Heim, R., McCaffery, J. M., Adams, J. A., Ikura, M., & Tsien, R. Y. (1997) Nature 388, 882-887.
2. Miyawaki, A., Griesbeck, O., Heim, R., & Tsien, R. Y. (1999) Proc Natl Acad Sci U S A 96, 2135-2140.
