# Supplementary material for: Small-molecule targeting of translation initiation for cancer therapy
Source: Oncotarget. 2013 Aug 14;4(10):1606–17. doi: 10.18632/oncotarget.1186 (PMC3858549; doi:10.18632/oncotarget.1186)
Supplement: Supplementary file 2 [file oncotarget-04-1606-s002.pdf]

Supplemental Table 1. Effect of anti-cancer agents (20 μM) on F-luc/R-luc ratio in the ternary complex assay.

Agent	Mechanism of Action	Relative Fluc/Rluc Ratio (+/- SEM)
Camptothecin	Topoisomerase inhibitor	1.3+0.3
Colchicine	Inhibitor of tubulin polymerization	1.2+0.3
Threo-1-phenyl	Glucolipid synthase inhibitor	1.5+0.4
Mitomycin C	Alkylating agent, DNA synthesis inhibitor	0.9+0.3
H-89	PK-A inhibitor	1.5+0.6
5-fluorouracil	Thymidilate synthase inhibitor	1+0.2
Epigallocatechin	Laminin Receptor 1 activation	
3-isobutyl-1-methylxanthine	Phoshodiesterase inhibitor	1.1+0.2
Dilthiazem	Ca++ channel blocker	1.4+0.4
Amiloride	Na+ channel blocker	1.1+0.3
Okadaic acid	Protein Phoshatase 1 inhibitor	1+0.3
Somatostatin	Inhibitor of growth hormone secretion	0.9+0.2
Glycyl-l-histidyl acetate	Not known	1+0.1
Etoposite	Topoisomerase I inhibitor	1+0.2
CLT	Ca^{++} store depletion	6 + 1.1
